# Large Fibroepithelial Polyp of Vulva

**DOI:** 10.1155/2011/273181

**Published:** 2011-11-24

**Authors:** Mahesha H. Navada, Poornima Ramachandra B. Bhat, Sujaya V. Rao, Nagarathna G.

**Affiliations:** Department of Obstetrics and Gynaecology, Father Muller Medical College, Karnataka, 575 002 Mangalore, India

## Abstract

A rare case of a large fibroepithelial polyp (FEP) of the vulva is described. The polypoidal growth was 10 cm in its largest diameter, having a long pedicle with features of inflammation secondary to infection, and was found arising from the left labia majora. The patient did not manifest any signs of recurrence following excision. A large and infected FEP of the vulva is a rare occurrence and hence reported.

## 1. Introduction

The fibroepithelial polyps (FEPs), which are also referred to as acrochordons or skin tags, are common lesions that typically occur in adults, especially obese women. They show a predilection for the neck, axillae, and groin. FEPs of the lower genital tract often develop in young to middle-aged women and present more commonly in the vagina.They are less common in the vulva and are rarely present in the cervix [1]. The polyps usually grow to a size of 1-2 cm and generally do not exceed this. Biopsy is often necessary to make a definitive diagnosis as their clinical features may overlap with those of malignant neoplasms [2, 3].

## 2. Case Report

**Figure 1:**
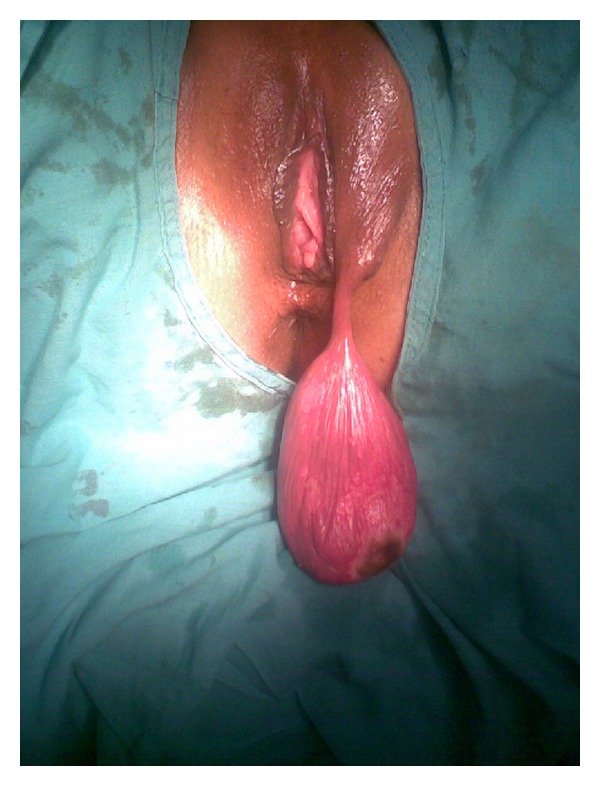
Large polypoidal growth from the left labia majora.

**Figure 2:**
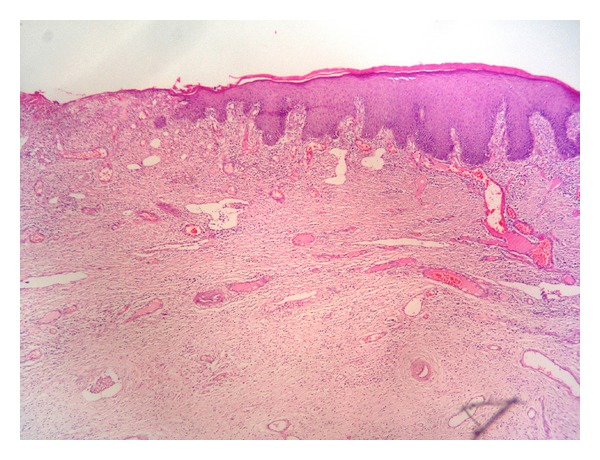
Fibrocollagenous stroma covered by stratified squamous epithelium with areas of ulceration.

A 27-year-old woman, para 4 living 4, presented with a swelling in the left labia, which was noticed 6 months back. There was a history of pain over the swelling and fever for 3 days. Her menstrual history was normal. She had previous vaginal deliveries and the last childbirth was one and half years back. Her build was average. The general physical examination and systemic examination were normal. Local examination revealed a large polypoidal growth covered by skin measuring 10 × 6 × 4.5 cm. arising from the left labia majora (Figure 1). The skin over the growth was ulcerated with signs of inflammation. The investigations revealed leukocytosis (WBC 13700/c mm) and an ESR of 70 mm (1st hour). Other laboratory investigations were normal including blood sugar. Total surgical excision of the mass was performed. Histopathological examination reported polypoidal tissue lined by stratified squamous epithelium with fibrocollagenous tissue in the subepithelium (Figure 2). The stroma containing blood vessels showed a mixed infiltrate of neutrophils and lymphocytes, suggestive of an infected fibroepithelial stromal polyp.

## 3. Discussion

FEPs of the vulval region are uncommon tumors. They usually arise in hair-bearing skin but may be found on the labia minora [1]. The origin is most probably from a regressing nevus [4]. These tumors vary in their clinical appearance from small fleshy colored or pigmented papillomatous growths resembling condylomata to large pedunculated tumors that are often hypopigmented. Histologically, FEPs may be of two types: one that is predominantly epithelial and the other that is primarily stromal. Frequent irritation seems to be an important causative factor, especially, in persons who are obese. An opinion also exists that FEPs are simply the effect of skin aging, with many factors responsible for their development. Hormone imbalances may facilitate the development of FEPs (e.g., high levels of estrogen and progesterone during pregnancy). Larger lesions are likely to arise from the proliferation of mesenchymal cells within the hormonally sensitive subepithelial stromal layer of the lower genital tract. Rarely, these stromal cells show marked atypia [1]. Infection is an unknown entity in FEP. The inflammation found in our case may be secondary to infection at the site of traumatic surface erosion.

In conclusion, large FEP of the vulval region is a rare benign tumor that can be misinterpreted as malignant owing to its wide range of morphological appearances. Expert pathological interpretation may be necessary to exclude atypical tumors and malignant neoplasms or to indicate proper treatment.

##  Consent

Consent to publish the images in this paper has been obtained from the patient.
